# Supplementary material for: Look-alike modelling in violence-related research: A missing data approach
Source: PLoS One. 2025 Jan 14;20(1):e0301155. doi: 10.1371/journal.pone.0301155 (PMC11731862; doi:10.1371/journal.pone.0301155)
Supplement: S1 Table — (DOCX) [file pone.0301155.s001.docx]

Supporting Information

S1 Table - Variable harmonising across the CSEW and RCEW

|  | CSEW | RCEW |
| --- | --- | --- |
| *Type of sexual violence (included in vector C)* | | |
| Rape | Rape (last 12 months)  Attempted Rape (last 12 months) | Assault by penetration  Voyeurism  Sexual assault by a trusted adult  Sexual assault by adult relative  Sexual assault by an adult  Sexual assault by a child family member  Sexual assault by a child  Sexual bullying  Penetration by object  Gang related sexual violence  Forced sexual activity in public  Exposed to sexual images  Sexual harassment  Serious sexual assault  Sexual violence  Sexual exploitation  Sexual assault |
| Other sexual violence and abuse | Indecent assault (last 12 months)  Serious wounding with sexual motive (last 12 months)  Other wounding with sexual motive (last 12 months) | \| Assault by penetration \| \| --- \| \| Exposed to sexual images \| \| Forced sexual activity in public \| \| Gang related sexual violence \| \| Penetration by object \| \| Sexual assault by a child \| \| Sexual assault by a child family member \| \| Sexual assault by a trusted adult \| \| Sexual assault by adult relative \| \| Sexual assault by an adult \| \| Sexual bullying \| \| Voyeurism \| |
| *Victim-perpetrator relationship (included in vector C)* | | |
| Domestic | Husband/ wife/ partner  Former husband/wife/partner  Current boyfriend/girlfriend  Former boyfriend/girlfriend  Son/daughter (in law)  (Ex) husband/(ex) wife/(ex) partner/(ex) boyfriend/(ex) girlfriend of someone else in household  Other household member | Current partner  Ex-partner  Relative |
| Acquaintance | Workmate/colleague  Client/members of public contacted through work  Neighbour  Tradesman/ builder/ contractor  Friend/acquaintance  Other | Acquaintance |
| Stranger or unknown | No relationship – Just seen them around  Young person from local area   Did not know perpetrator | Stranger |
| *Physical health impact* | | |
| No injury | No force or violence used.  Not bruised, scratched, cut or injured in any way | If no injuries listed in incidentimpact so coded as ‘0’ in physicalhealth_impact |
| Injury | Bruised, scratched, cut or injured in any way. | Memory loss  Physical injuries  Body problems  Gynae disorder  Sexually transmitted infection |
| *Gender* | | |
| Man | Male (Q: Is (name) male or female?) | Man  Transgender male |
| Woman | Female (Q: Is (name) male or female?) | Woman  Transgender female |
| *Relationship status* | | |
| Married/Cohabiting | Married  In a registered same-sex civil partnership  Yes (Q: living with someone in this household as a couple?) | Civil partnership  Cohabiting  Married |
| Single/non-resident relationship/Widowed | Single, that is, never married and never registered in a same-sex civil partnership  Widowed  Surviving partner from a same-sex civil partnership | In a relationship  Single  Widow  Widower |
| Separated/Divorced | Separated, but still legally married  Divorced  Separated, but still legally in a same-sex civil partnership  Formerly in a same-sex civil partnership which is now legally dissolved | Divorced  Separated |
| *Ethnicity* | | |
| *White* | English/Welsh/Scottish/Northern Irish/British  Irish  Gypsy or Irish Traveller  Any other white background | Albanian  White British  White Cornish  White Eastern European  White English  White European  White Irish  White Northern Irish  White Other  White Scottish  White Welsh  White Western European  White  White African  Turkish  Turkish or Turkish Cypriot  Traveller Irish heritage  Polish  Portuguese  Polish  Italian  Greek  Greek Cypriot  Gypsy or Roma  Bosnian Herzegovinian  Any other White background |
| *Not White* | Indian  Pakistani  Bangladeshi  Chinese  Any other Asian background  African  Caribbean  Any other Black / African / Caribbean background  White and Black Caribbean  White and Black African  White and Asian  Any other Mixed / Multiple ethnic background  Arab  Any other ethnic group | Asian British  Asian or Asian British  Bangladeshi  Chinese  Filipino  Indian  Mauritian  Pakistani  Thai  Vietnamese  Any other Asian background  Black African  Black British  Black Caribbean  Black European  Black or Black British  Any other Black background  Mixed or dual  White and Asian  White and Bangladeshi  White and Black African  White and Black Caribbean  White and Indian  Any other Mixed background  Moroccan  Latin/south/central American  Kurdish  Iranian  Egyptian  Arab other  Any other ethnic group |
| *Employment status* | | |
| Employed | Yes (Q: Did you do any paid work in the seven days ending Sunday the [date], either as an employee or as self-employed?)  Yes (Q:Did you have a job or business you were away from?) | Apprentice  Employed  Employed full-time  Employed part-time  Self-employed  Self-employed full-time  Self-employed part-time |
| Unemployed | Yes (Q: Thinking of the FOUR WEEKS ending Sunday the [date], were you looking for any kind of paid work or government training scheme at any time in those 4 weeks?) | Unemployed  Registered unemployed  NEET  Homemaker |
| Outside labour force | Yes (Q: Did you do any UNPAID work in that week (ending Sunday the [date]) for any business that you own? Or (any UNPAID work for a business) that a relative owns?)  Looking after the family/home  Temporarily sick or injured  Long-term sick or disabled  Retired from paid work  Any other reason [any other reason not being employed or looking for work] | Carer  Carer full-time  Carer part-time  Long term sick  Registered disabled  No recourse  Retired |
| Student | Student  Were you on a government scheme for employment training in that week (ending Sunday the [date])? | Student  Student full-time  Student part-time |
| *Dependents (number of children)* | *Numerical* | None  One  Two  Three  Four  Five or more |
| *Housing Tenure* | | |
| Homeowner/lives in owned home | Own it outright  Buying it with the help of a mortgage or loan  Pay part rent and part mortgage (shared ownership) | Homeowner  Living with parents  With family  With friends |
| Renter | Rent it | Private rent |
| Other | Live here rent free (inc. rent free in relative/friend's/squatting) | B&B  Foster care  General hospital  HMO  Homeless  Hostel  Housing association  In care  Local authority  Mental health unit  Mobile home  Mother and baby unit  Other  Prison  Refuge accommodation  Residential  Service family accommodation  Settled accommodation  Sheltered accommodation  Sofa surfing  Student accommodation  Supported housing  Temp accommodation  Tent |
